# Worldwide evidence about infant stunting from a public health perspective: A systematic review

**DOI:** 10.7705/biomedica.6017

**Published:** 2021-09-22

**Authors:** Paola Rueda-Guevara, Natalia Botero-Tovar, Kenny Margarita Trujillo, Andrea Ramírez

**Affiliations:** 1 Salud Poblacional, Fundación Santa Fe de Bogotá, Bogotá, D.C., Colombia Fundación Santa Fe de Bogotá BogotáD.C Colombia; 2 Nutrición Social, Bogotá, D.C., Colombia Nutrición Social BogotáD.C Colombia; 3 Facultad de Medicina, Universidad de los Andes, Bogotá, D.C., Colombia Universidad de los Andes Facultad de Medicina Universidad de los Andes BogotáD.C Colombia

**Keywords:** Child development, failure to thrive, malnutrition, intersectoral collaboration, health status indicators, primary prevention, desarrollo infantil, insuficiencia de crecimiento, desnutrición, colaboración intersectorial, indicadores de salud, prevención primaria

## Abstract

**Introduction:**

According to the World Health Organization (WHO) global estimates for 2017, 9.6% of children under 5 years old are stunted. Worldwide evidence shows that actions for preventing stunting and catching-up growth are relevant if addressed by all the sectors involved. Therefore, there is a need to identify 'intersectoral actions' to address the risk of stunting during pregnancy and the first 2 years of life.

**Objective:**

To identify and describe worldwide evidence for prevention, nutritional interventions, and 'intersectoral collaboration' efforts against stunting in infants.

**Materials and methods:**

We conducted a systematic review in 2019 (PROSPERO CRD42019134431). The search included PubMed, OVID, and Web of Science, as well as WHO and the Food and Agriculture Organization of the United Nations (FAO) official documents and expert recommendations.

**Results:**

We selected 231 studies: 86.1% described prevention-related factors, 30.7%, nutritional interventions, and 52.8% intersectoral collaboration efforts; 36.4% of the studies were conducted in multiple regions; 61% of the studies described the importance of interventions during pregnancy, 71.9% from birth up to 6 months old, and 84.8% from 6 months up to 2 years old. The most frequent variables described were antenatal care, nutritional counseling for the mother and the newborn, and counseling on micronutrient supplementation.

**Conclusions:**

Evidence-based understanding of actions geared towards monitoring the risk of stunting-associated factors from pregnancy up to 2 years old is critical.

## Resumen

**Introducción.:**

Según estimaciones mundiales de la Organización Mundial de la Salud (OMS) para el 2017, el 9,6 % de los niños menores de cinco años padecen retraso del crecimiento. La evidencia mundial ha demostrado que las acciones para prevenir el retardo del crecimiento o para tratarlo adquieren relevancia si las abordan todos los sectores involucrados. Por lo tanto, es necesario determinar las acciones intersectoriales para atender el riesgo de retraso del crecimiento durante el embarazo y los dos primeros años de vida.

**Objetivo.:**

Rastrear y describir la evidencia mundial para la prevención, las intervenciones nutricionales y los esfuerzos de colaboración intersectorial contra el retraso del crecimiento en los lactantes.

**Materiales y *métodos*:**

Se hizo una revisión sistemática en el 2019 (PROSPERO CRD42019134431). La búsqueda incluyó PubMed, OVID y Web of Science, así como documentos oficiales de la OMS y la Organización para la Agricultura y la Alimentación de Naciones Unidas (FAO) y recomendaciones de expertos.

**Resultados.:**

Se seleccionó un total de 231 estudios: 86,1 % describían factores relacionados con la prevención, 30,7 %, intervenciones nutricionales, y 52,8 %, esfuerzos de colaboración intersectorial; 36,4 % de ellos se llevaron a cabo en múltiples regiones. Del total, el 61 % de los estudios se enfocaba en la importancia de las intervenciones durante el embarazo, el 71,9 % desde el nacimiento hasta los seis meses y el 84,8 % desde los seis meses hasta los dos años. Las variables descritas con mayor frecuencia fueron la atención prenatal, el asesoramiento nutricional para la madre y el recién nacido, y el asesoramiento sobre la suplementación con micronutrientes.

**Conclusiones.:**

La comprensión basada en la evidencia de las acciones orientadas a monitorear el riesgo de factores asociados al retraso del crecimiento desde el embarazo hasta los dos años, es fundamental.

Stunting is a form of malnutrition. It is an important limitation to human development during the first crucial 1,000 days of life from pregnancy to 2 years of age. According to the World Health Organization (WHO), stunting affects around 162 million children under the age of 5 globally. Stunting in 0 to 59 month-old infants is defined as a height/length-for-age less than two standard deviations (-2 SD) below the median of reference ^1^^,^^2^. According to WHO global estimates for 2017, 9.6% of children under 5 are stunted ^3^. Furthermore, projections indicate that 127 million children in this age group around the world will be stunted by 2025. Thus, intersectoral actions are necessary to accomplish the 2025 target of a 40% global reduction in the number of stunted children under 5 ^1^.

Several studies have shown that stunting has negative effects on the life course of children from pregnancy to adulthood with short-term consequences such as increased susceptibility to infectious diseases like acute diarrheal disease and acute respiratory infection both of which contribute to infant mortality ^4^. Studies have also shown that stunting can affect learning potential preventing adequate cognitive development and limiting learning capacity ^5^, which in turn increases the probability of late incorporation to school, school failure, dropout, and low academic performance ^6^. Stunting can lead to the loss of 2 or 3 years of schooling and a subsequent income reduction of 23% in adulthood compared to children with adequate growth ^7^. An association with an increased risk of chronic non-communicable diseases such as diabetes, hypertension, and cardiovascular disease has also been reported ^8^. As for national development, stunting reduces the gross domestic product by up to 3% ^9^.

Based on the available evidence on highly cost-effective actions in the prevention and management of stunting, various measures have been proposed to reduce the irreversible physical and cognitive effects of the deficit in height gain in the first years of life. Bhutta, *et al.* demonstrated the potential effect of 10 specific nutritional actions on children's health that could limit growth and severe acute malnutrition. Examples of the interventions that could reduce stunting by 20.3% if scaled up to a 90% coverage include folic acid supplementation in the preconception period, maternal food supplementation, maternal calcium supplementation, maternal multiple micronutrient supplementation, and the promotion of breastfeeding, among others ^10^.

Worldwide evidence shows that these actions for the prevention of stunting and promotion of catch-up growth are relevant if addressed by all the sectors involved. The WHO defines intersectoral actions as:

a recognized relationship between part or parts of the health sector and part or parts of another sector, that has been formed to take action on an issue or to achieve health outcomes in a way that is more effective, efficient or sustainable than could be achieved by the health sector working alone. ^1^

Therefore, there is a need to identify actions under way to monitor prevalence, nutritional interventions, and intersectoral collaboration to address the risk of stunting during pregnancy and the first 2 years of life. Prevention of and interventions against stunting and contributing risk factors depend upon the identification of those actions that improve a child's age-related growth and height/length gain based on an inter-sector coordination approach ^11^. Through a systematic review of the literature, we aimed at identifying and describing evidence about infant stunting prevention, associated factors, nutritional interventions, and intersectoral collaboration efforts worldwide.

## Materials and methods

We conducted a systematic review following the Preferred Reporting Items for Systematic Reviews and Meta-Analyses (PRISMA) guidelines registered at the International Register of Systematic Prospective Reviews (PROSPERO) and approved under registration number CRD42019134431.

### Search strategy

The search was conducted on February 27, 2019, in MEDLINE (using the free access tool PubMed® and OVID) and Web of Science. The search terms were "Malnutrition/prevention & control*" OR stunting, AND "infant" OR "child" AND "prevention" OR "treatment" OR "intervention" OR "primary care" selected from the Medical Subject Headings (MeSH) and Health Sciences Descriptors (DeCS). Terms could be found anywhere in the article, title, or abstract. We refined the search by including the term "birth-23 months.

We also conducted a search of gray literature to identify the evidence on intersectoral collaboration and stunting. We searched in MEDLINE (using the free access tool PubMed® and OVID), Web of Science, and Google for eligible studies using the terms Malnutrition/prevention & control^*^ OR stunting OR undernutrition AND public-private OR Public Health^*^ OR Public Sector^*^ OR Public-Private Sector Partnerships^*^ OR inter-sectoral OR multi-sectoral AND partnerships OR collaboration OR consolidation OR cooperation OR planning AND infant OR child. We searched as well secondary data sources such as the WHO, FAO, World Bank, and the United Nations International Children's Emergency Fund (UNICEF) websites to find eligible documents using the terms "stunting" and/or "chronic undernutrition."

We did not restrict the search by language or year of publication. The search equations used for each of the databases are included in Supplementary document 1. We used the EndNote X7 software to manage references, remove the duplicates, and obtain full documents to review.

### Inclusion and exclusion criteria

Original articles were included using the following criteria: (a) any study on stunting prevention, associated factors, or nutritional interventions; (b) any study on stunting intersectoral collaboration; (c) evidence on interventions was restricted to infants (children under 2 years old), and (d) documents on public health policies, national guidelines, infographics, systematic reviews, meta-analyses, observational studies, and experimental studies. No language or country restrictions were applied.

Exclusion criteria included: (a) clinical studies; (b) interventions in children older than 2 years, and (c) studies that did not explain intersectoral collaboration among sectors, or that were not oriented towards stunting prevention or catch-up growth.

Three researchers conducted the screening process independently (AR, NB, and PR), applying pre-established inclusion and exclusion criteria to select studies for complete reading; they also conducted the extraction. The senior researcher (AR) solved doubts while disagreements were resolved through consensus.

### Data extraction

The three independent researchers extracted the data from the studies using a standardized data extraction format. Characteristics included: (a) publication (title, author[s], journal, year, study site [country]); (b) study characteristics (design, sample size); (c) stunting prevention, nutritional interventions, or intersectoral collaboration; (d) evidence on health, social assistance, caregivers' education, community empowerment for stunting prevention, catch-up growth, or intersectoral collaboration. We grouped the study countries according to WHO regions.

### Variables of interest

The main outcome was infant stunting defined as height/length-for-age at least two standard deviations below the median of reference.

Independent variables included stunting-associated protective and risk factors, prevention strategies, nutrition interventions, and intersectoral collaboration along the life course. Table 1 shows study characteristics by stunting prevention or management strategies. Data syntheses were done for the following topics: (a) Poor nutrition during pregnancy and the first 2 years of life; (b) lack of breastfeeding; (c) poor complementary feeding; (d) micronutrient supplementation; (d) poor health services for antenatal care; (e) child development and growth consultations including height-forage assessment and documentation of risk or chronic undernutrition in children under 2; (f) community, parent, and caregiver education on chronic undernutrition in children under 2, and (g) social and health workers training on chronic undernutrition in children under 2.

**Table 1:** Characteristics of the studies included according to the strategies to prevent or treat stunting by life-course

	Prevention		Interventions		Intersectoral collaboration	
	n	%	n	%	n	%
Pregnancy	111	48	26	11	80	35
Health attention	101	44	18	8	61	26
Social attention	37	16	5	2	49	21
Health education or counselling	45	19	7	3	30	13
Empowerment community	36	16	10	4	49	21
Up to 6 months old	135	58	51	22	92	40
Health attention	106	46	32	14	68	29
Social attention	45	19	10	4	51	22
Health education or counselling	65	28	25	11	37	16
Empowerment community	46	20	15	6	53	23
Up to 1 year old	166	72	55	24	115	50
Health attention	142	61	35	15	86	37
Social attention	65	28	14	6	61	26
Health education or counselling	87	38	22	10	45	19
Empowerment community	57	25	17	7	75	32

N=231

### Study quality evaluation

Study quality was measured according to the checklist proposed by Downs and Black (1998). We evaluated reporting, external validity, internal validity bias, internal validity confounding, and power of studies based on 27 items if applicable (Supplementary table 1). Reviewers independently assessed the risk of bias while disagreements were resolved through consensus.

### *Data analysis*

Descriptive statistics (i.e., frequencies, means, ranges, and percentages) were used to report the variables of interest. Table 2 shows the characteristics of the documents included in the review.

**Table 2:** Characteristics of the studies included in the systematic review

			Prevention		Interventions		Intersectoral collaboration	
	n	%	n	%	n	%	n	%
Total studies	231		199	86.1	71	30.7	122	52.8
World Health Organization region covered								
The Americas-PAHO	28	12.1	26	13.1	6	8.5	11	9.0
Africa-AFRO	51	22.1	41	20.6	18	25.4	21	1 7.2
Western Pacific-WPRO	15	6.5	14	7.0	4	5.6	6	4.9
Europe-EURO	2	0.9	2	1.0	1	1.4	0	0.0
Eastern Mediterranean-EMRO	7	3.0	3	1.5	2	2.B	4	3.3
Southeast Asia-SEARO	44	19.0	39	19.6	20	28.2	22	18.0
Multiple regions	84	36.4	74	37.2	20	28.2	58	47.5
Study design								
Cross sectional	54	23.4	53	98.1	18	35.3	25	46.3
Cohort	7	3.0	6	B5.7	4	57.1	1	14.3
Case control	2	0.9	1	50.0	0	0.0	2	100.0
Intervention	53	22.9	45	84.9	18	34.0	15	28.3
Systematic review	10	4.3	10	90.9	5	45.5	3	27.3
Theoretical and conceptual document	65	28.1	56	86.2	20	30.8	46	70.8
Qualitative study	12	5.2	9	75.0	3	25.0	8	66.7
Other: Health policies, infographics, national guidelines	27	11.7	20	76.9	4	15.4	23	88.5
Lifecycle stages								
Pregnancy	141	61.0	112	79.4	25	17.7	80	56.7
Birth to 6 months	166	71.9	137	82.5	50	30.1	93	56.0
6 months to 24 months	196	84.8	165	84.2	55	28.1	116	59.2

## Results

### Data synthesis

**Figure 1:**
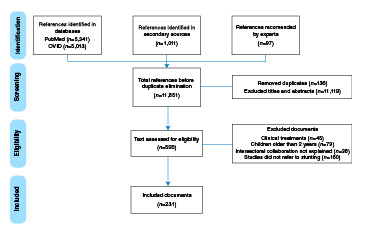
Flowchart of studies selection

Note: Taken from PRISMA (2009)

The search strategy resulted in 11,851 titles to examine. After removing duplicates (n=136 documents) and excluding 11,119 by title and abstracts, a list of 596 unique citations was assessed for eligibility. We identified 231 articles for full-text review (figure 1).

The characteristics of the documents included in the review are presented in table 2. Most of the studies were conducted in multiple regions (n=84), followed by those in Africa (n=51) and Southeast Asia (n=44). The most frequent topics were stunting prevention (n=199), intersectoral collaboration (n=122), and nutritional interventions (n=71). Regarding the type of study, interventions were the most frequent (n=53), followed by cross-sectional (n=51), cohort (n=7), and cases and controls (n=2) studies. The sample size of the observational studies ranged from 48 to 699,686 participants. Data also included systematic reviews (n=10), qualitative studies (n=12), theoretical and conceptual documents (n=65), and health policies, infographics, and national guidelines (n=27).

Among life-course risk factors and interventions to prevent stunting, the most frequent was from 6 to 24 months (n = 196), followed by birth to 6 months (n=166), and pregnancy (n=141). We present here the results on stunting prevention, nutritional interventions, and intersectoral collaboration efforts evidenced in the literature. Table 3 shows study characteristics and life-course stages from pregnancy up to 24 months by region.

**Table 3:** Prevention of stunting in children under 2 years of age by life-course stage

	N = 231		AFRO (1)		EMRO		EURO (3)		PAHO (4)		SEARO (5)		WPRO (6)		Multiple regions	
	n	%	n	%	n	%	n	%	n	%	n	%	n	%	n	%
Pregnancy	141	61.0	24	17.0	4	2.8	0	0.0	15	10.6	33	23.4	7	5.0	58	41.1
Antenatal care	33	23.4	2	6.1	1	3.0	0	0.0	7	21.2	8	24.2	4	12.1	11	33.3
Education or counseling in nutrition for mothers and newborns	45	31.9	8	1 7.8	1	2.2	0	0.0	1	2.2	12	26.7	3	6.7	20	44.4
Mother's height	11	7.8	1	9.1	0	0.0	0	0.0	1	9.1	4	36.4	0	0.0	5	45.5
Micronutrient supplements	55	39.0	5	9.1	0	0.0	0	0.0	6	10.9	13	23.6	4	7.3	27	49.1
Adequate weight gain	15	10.6	4	26.7	0	0.0	0	0.0	0	0.0	3	20.0	1	6.7	7	46.7
Birth until 6 months of age	166	71.9	29	17.5	4	2.4	1	0.6	17	10.2	33	19.9	14	8.4	68	41.0
Low birth weight	19	11.4	3	15.8	0	0.0	0	0.0	3	15.8	3	15.8	2	10.5	8	42.1
Low height for age	6	3.6	0	0.0	0	0.0	0	0.0	1	16.7	2	33.3	1	16.7	2	33.3
Breastfeeding practices	71	42.8	9	12.7	3	4.2	0	0.0	6	8.5	16	22.5	3	4.2	34	47.9
Counseling in breastfeeding and nutrition	62	37.3	11	1 7.7	1	1.6	0	0.0	4	6.5	12	19.4	3	4.8	31	50.0
Micronutrient supplements	33	19.9	6	18.2	1	3.0	0	0.0	3	9.1	5	15.2	5	15.2	13	39.4
Early detection of infants at risk or with low HAZ	17	10.2	1	5.9	0	0.0	1	5.9	3	1 7.6	6	35.3	1	5.9	5	29.4
Growth and development control	33	19.9	4	12.1	0	0.0	1	3.0	6	18.2	8	24.2	2	6.1	12	36.4
Vaccines	21	12.7	3	14.3	2	9.5	0	0.0	1	4.8	4	19.0	2	9.5	9	42.9
6 to 24 months of age	196	84.8	37	18.9	6	3.1	2	1.0	22	11.2	36	18.4	13	6.6	80	40.8
Complementary feeding	100	51.0	16	16.0	2	2.0	1	1.0	6	6.0	19	19.0	7	7.0	49	49.0
Counseling in nutrition	83	42.3	15	18.1	1	1.2	1	1.2	5	6.0	12	14.5	7	8.4	42	50.6
Breastfeeding	52	26.5	8	15.4	1	1.9	0	0.0	5	9.6	12	23.1	3	5.8	23	44.2
Micronutrient supplements	72	36.7	13	18.1	2	2.8	0	0.0	8	11.1	15	20.8	5	6.9	29	40.3
Deworming	12	6.1	2	16.7	0	0.0	0	0.0	1	8.3	3	25.0	1	8.3	5	41.7
Early detection of infants at risk or with low HAZ	17	8.7	3	1 7.6	0	0.0	0	0.0	3	1 7.6	4	23.5	3	1 7.6	4	23.5
Growth and development control	41	20.9	8	19.5	0	0.0	1	2.4	7	1 7.1	8	19.5	4	9.8	13	31.7
Vaccines	23	11.7	3	13.0	1	4.3	0	0.0	1	4.3	3	13.0	4	1 7.4	11	47.8

HAZ: Height for age

### Stunting prevention

Out of the studies reviewed, 199 discussed stunting prevention factors and were mainly documented in Africa (n=41), Southeast Asia (n=39), and the Americas (n=26) (table 2).

Table 3 shows the evidence-supported strategies for stunting prevention during the window of opportunity known as the first 1,000 days of life, from gestation up to 2 years old. The topics mostly addressed in studies discussing stunting prevention during pregnancy were micronutrient supplements (n=55); education or counseling in nutrition for mothers and newborns (n=45); antenatal care (n=33) with more than 23.6% of the evidence originated in Southeast Asia, and adequate maternal weight gain (n=15) Africa being the region with the highest number of strategies focusing on adequate gestational weight-gain to prevent stunting. The least-reported topic was maternal height (n=11). From birth up to 6 months, studies on stunting prevention mostly focused on the following topics: breastfeeding practices (n=71); counseling on breastfeeding and nutrition (n=62), and growth and development control (n=33) again with the largest amount of evidence originated in Southeast Asia. The least-reported topic was low height for age (n=6). Finally, studies discussing stunting prevention from 6 to 24 months were mostly concerned with the following topics: complementary feeding (n=100); counseling in nutrition (n=83), and micronutrient supplements (n=72) with the largest amount of studies conducted in Africa.

### Nutritional interventions to address stunting and achieve catch-up growth

Seventy-one of the studies reported evidence on nutritional interventions to address stunting (table 2); those discussing catch-up growth were mainly documented in Southeast Asia (n=20), Africa (n=18), and the Americas (n=6). Studies on nutritional interventions during pregnancy were mostly concerned with micronutrient supplements (n=55) and antenatal care (n=33). From birth to 6 months, studies were mostly concerned with breastfeeding practices (n=71) and micronutrient supplements (n=33) while from 6 to 24 months they were mostly focused on complementary feeding (n=100) and micronutrient supplements (n=72).

In addition, 122 studies reported on intersectoral collaboration for stunting prevention and nutritional interventions. Intersectoral collaboration studies were mainly from Southeast Asia (n=22), Africa (n=21), and the Americas (n=11). It was also evident in these studies that intersectoral actions during pregnancy, in children under 6 months and under 2 years are necessary for stunting prevention. It was also evident in these studies that intersectoral actions during pregnancy in children under 6 months and under 2 years are necessary for stunting prevention (Table 4).

**Table 4:** Intersectoral collaboration to address stunting or its risks by sectors.

Agriculture	Improve nutrition messages by coordination between community workers from the health and agriculture sectors Guarantee food security
Health	Implement antenatal control to prevent stunting Surveillance of linear growth and development Guarantee micronutrient supplementation Develop programs for adequate breastfeeding practices in coordination with the social assistance sector
Education	Develop health literacy and nutrition knowledge Develop strategies to reduce adolescent pregnancy and promote higher education and women’s empowerment
Social assistance	Implement conditional cash transfers to increase the use of health services and stunting surveillance Offer nutritional complementation for vulnerable homes with food insecurity
Economic development	Promoting community workers for stunting surveillance
Government	Integrate nutrition into the national development agenda Develop national nutritional policies and/or dietary guidelines Implement policies for the protection, promotion, and support of optimal breastfeeding practices Ensure a common understanding between sectors regarding the severity of stunting Ensure a mechanism for local delivery of nutrition services Promote and support local ownership of nutrition programs and their outcomes Monitor intersectoral collaboration with indicators at national, provincial, and district levels
Private	Participate in public-private partnerships aligned with policies to improve child growth and development Integrate nutrition in company policies Provide experience in communication and financial support for a communication plan aimed at reducing stunting

## Discussion

This systematic review of the literature is the first one to identify and describe stunting prevention strategies, associated factors, nutritional interventions, and intersectoral collaboration in children up to 2 years of age worldwide. It is useful for informing public health and policy decision-makers. Key findings indicated that:

- Most stunting prevention strategies, nutritional interventions, and intersectoral collaboration were conducted in low- and middle-income countries, mainly in the Africa (AFRO), Southeast Asia (SEARO), and the Americas (PAHO) WHO regions.
- The evidence on stunting prevention in children up to 2 years by life-course stage and region showed the need to wage actions to strengthen the design and implementation of nutritional interventions.
- The specific interventions for catch-up growth were the least prevalent topic in this review.

There are knowledge gaps on intersectoral coordination for stunting prevention and/or intervention.

The strengths of this study are twofold: First, we complemented the collection and analysis of the most updated and high-quality data available with the revision of WHO, PAHO, and FAO official documents, as well as expert recommendations. Second, we characterized the evidence according to the life course from pregnancy to 2 years of age focused on prevention strategies, nutritional interventions, and intersectoral collaboration against stunting.

It has been suggested that stunting is an accurate indicator of inequalities in human development and that height-for-age is the best indicator of children's well-being ^12^. The evidence found in this systematic review came mostly from the AFRO, SEARO, and PAHO regions where most interventions were conducted. Our findings show a region-specific description of actions to address stunting, where relevance and use depend on the country-specific context. Stunting tendencies from 1990 to 2012 showed that the number of stunted children under 5 was halved in Asia and the Latin America/Caribbean region, but its burden in Africa increased by 24% ^1^. As various authors have argued, factors contributing to stunting correlate with poverty and socioeconomic development ^13^^-^^16^. Black, *et al.,* for example, concluded that in most countries, the prevalence of stunting among children under 5 was 2.5 times higher in the low-wealth quintile compared with the highest wealth quintile ^8^. Moreover, studies have demonstrated that a one-dollar increase in the *per capita* GDP was associated with a 0.003-unit decrease in stunting (p<.001) ^17^ and previous research using these platforms concluded that low stunting magnitudes were found in low- and middle-income countries with strong nutrition governance ^17^, which might be due to the existence of an intersectoral mechanism to address nutrition, the existence and adoption of a nutritional strategic plan, and national nutritional policies and/or dietary guidelines. These programs counted with resource allocation for the national nutrition plan, strategies, or policies, as well as the inclusion of nutrition monitoring and surveillance components in health budgets ^17^.

Critical sectors mentioned for stunting interventions were agriculture, health, education, social assistance programs, employment, and government ^18^. Several studies identified intersectoral coordination between health and other sectors. As an example of coordination between health and social sectors, conditional cash transfers were found to increase the use of health services and stunting surveillance ^19^ while social assistance programs allowed nutritional complementation for vulnerable homes with food insecurity ^20^ and were considered helpful to reduce some of the limitations of families to invest optimally in their children. Such programs improved parents' knowledge about ideal practices to promote parenting and, also, had large impacts on parental behaviors and children's development in various contexts.

In some cases, social assistance programs including nutrition-specific objectives aligned with local needs and health services to increase access to food and nutritional supplements ^21^. Health literacy and nutrition knowledge were documented as activities where different sectors could participate and government actions geared towards the promotion of breastfeeding ^20^. Researchers also evidenced coordination between community workers from the health and agriculture sectors to improve nutrition messages ^22^.

As for the evidence on stunting prevention by life-course, stage, and region, studies have shown that actions are required to strengthen the design and implementation of nutritional interventions. Stunting often begins in utero, according to India's National Family Health Survey 2005-2006 ^12^. Antenatal control constitutes one of the most important factors to reduce stunting risk, as a pregnant woman who has at least one antenatal control has a 4.11% reduction in stunting odds ^23^. Antenatal control is also a critical action to control adequate weight gain during pregnancy; its inadequacy is a strong predictor of low birth weight and other conditions related to malnourishment ^23^.

The importance of adequate weight gain to prevent stunting was documented in pregnant women in Mozambique in the framework of research coordinated by the Ministries of Health and Women and Social Action. These recommendations are supported by scientific evidence and, furthermore, they are inexpensive and easy to implement at the primary healthcare level in any country. Micronutrient supplementation for pregnant women (i.e., iron and folic acid) has been found to prevent micronutrient deficiencies and contribute to linear growth improvement in many studies ^20^^,^^24^^-^^26^.

A prevalent topic was adequate breastfeeding practices, particularly exclusive breastfeeding, during the first 6 months of life. Global strategies, such as Scaling Up Nutrition, 1000 Days, the Zero Hunger Challenge, and the Nutrition for Growth Summit, have demonstrated that early introduction of breastfeeding in the first hour after birth is a determinant factor for stunting prevention ^27^^,^^28^. Individual or group-based counseling sessions to promote exclusive breastfeeding were reported as important interventions to scale up ^29^. Other studies described the importance of the protection, promotion, and support of optimal breastfeeding practices, such as the initiation of breastfeeding within 1 hour of birth, exclusive breastfeeding counseling, lay support for breastfeeding through community-based and facility-based contacts, and additional control of the marketing of breastfeeding substitutes ^30^.

Other important interventions, such as the surveillance of low birth weight and low birth height/length, were important for the early detection of infants at risk, as were low height for age, vaccination status, and close growth monitoring ^9^. Maternal health literacy and education ^31^^,^^32^, as well as family nutrition practices based on the WHO infant and young child feeding guidelines ^33^, prevented stunting.

Researchers have acknowledged the difficulties associated with the diagnosis of stunting despite the global consensus on how to define and measure it ^12^. In some communities, short stature is the norm and linear growth is not routinely assessed in primary healthcare settings ^12^. In this context, surveillance of stunted infants is critical because they are seemingly healthy. For this reason, the literature supports raising awareness and informing caregivers that short stature is a nutrition-related problem and linear growth is associated with adequate care ^34^.

Interventions to address stunting are multifaceted ^30^; they include breastfeeding education and counseling, appropriate complementary feeding, micronutrient supplementation of pregnant women, nutrition counseling during pregnancy, vitamin A supplementation in infants and children 6 to 59 months of age, and zinc supplementation in the management of diarrhea, which improves nutrient uptake ^35^. These interventions often intertwine and stunting prevention messages take precedent over the specific catch-up growth messages ^13^.

The evidence synthesis in this systematic review clearly showed that a determinant factor in catch-up growth after a period of stunting was complementary feeding. Appropriate food introduction complementary to breastfeeding was relevant for catch-up growth with indicators such as minimum dietary diversity, minimum meal frequency, and minimum acceptable diet ^26^^,^^36^^,^^37^. Intensive counseling and emphasis on dietary diversity together with the promotion and consumption of animal source foods are successful interventions to improve complementary feeding ^10^^,^^38^.

Community workers and community participation were key to identifying pregnant women suffering from or at risk of food insecurity and helped to ease their route to health and social services; consequently, they facilitated the identification of adequate interventions for stunted children ^39^^,^^40^. It has also been suggested that time constraints, maternal depression, lack of family support, and breastfeeding while working should all be taken into account and monitored in the surveillance of stunting or the risk thereof ^28^.

As for intersectoral collaboration, the evidence highlighted a call to action from different sectors, but more is needed to develop indicators for the monitoring and evaluation of these partnerships. Linear growth improvement was possible through the national coordination of child and maternal nutrition services ^27^. Evidence also showed that coordination should be clearly defined specifying what should be coordinated, how, and by whom ^41^. Plans to evaluate intersectoral coordination should also be considered ^42^. In Peru, establishing political commitment, cooperation, and coordination was acknowledged as a social, economic, and health challenge crucial for reducing stunting ^43^. Mozambique stands out as an exceptional example, as specific roles and indicators were assigned to the health, social assistance, and educational sectors ^44^. The goal was to qualify advisors in healthy feeding and nutrition from each of these sectors and monitor the partnerships with indicators at national, provincial, and district levels ^44^. The literature also highlighted the importance of community mobilization and motivation to undertake initiatives for stunting prevention at the household level and to use services that improve child growth and development ^3^.

Sectors other than health have responsibilities in prevention and nutritional interventions against stunting. The education sector can develop strategies to reduce adolescent pregnancy and promote higher education and women's empowerment, for example ^15^^,^^45^^,^^46^.

The economic development sector can aid the health sector by promoting community workers for stinting surveillance ^27^; the communication sector is important for surveillance and intersectoral collaboration ^27^; studies also supported the notion that the academic sector should share the responsibility of integrating the knowledge on breastfeeding and complementary feeding and their promotion ^41^. The private sector can contribute its experience in communications and financial support to develop plans aimed at reducing stunting at the national, provincial, and district levels ^42^^,^^44^ as shown by the evidence on its relevance for food fortification in alignment with government policies ^27^^,^^42^^,^^47^. Successful intersectoral collaboration strategies for the prevention of stunting and nutritional interventions to address it suggest that multiple sectors can target this public health problem by raising public awareness.

Thus, the recommendations from this review are applicable at multiple levels. Governments should strengthen national information and surveillance systems to produce data to support policy-makers response to nutrition priorities in a timely fashion. Indicators on stunting risks and their prevention, nutritional interventions, and intersectoral collaboration should all be included in the surveillance systems. Policymakers should raise awareness and train health workers on the importance of antenatal control and counseling regarding adequate weight gain during pregnancy. Lastly, more research is needed on epidemiologic surveillance indicators for the monitoring of prevention and catchup growth in infants from an intersectoral collaboration perspective.

Stunting is a worldwide concern and one of the Sustainable Development Goals to be accomplished in the short term. Worldwide evidence about prevention strategies, nutritional interventions, and intersectoral collaboration to address stunting and its risk factors highlights the need to strengthen vigilance and public health interventions. Linear growth is the best indicator of overall child development during the first years of life and human development inequalities. Efforts to assess the available information are necessary to create epidemiologic surveillance indicators, which provide guidance and individualized support to adopt public policies addressed to this problem and encourage the involvement of multiple sectors. All countries should promote the implementation of stunting surveillance and the construction of structured and standardized indices for international comparability and monitoring.

There were some limitations in the study. The comprehensive search terms used could reduce some relevant studies as gray literature. The gray literature may have contained additional information that was not included, and, finally, we gathered the information from multiple and heterogeneous sources, which represents a limitation for data standardization in specific variables due to different measurement methods.

## Supplementary files

### Evaluation of study quality according to Downs and Black

**Table:** 

	Yes (n)	%	No (n)	%	Does not apply (n)	%
Reporting						
1. Is the hypothesis/aim/objective of the study clearly described?	228	98.7	2	0.9	0	0.0
2. Are the main outcomes to be measured clearly described in the Introduction or Methods section?	225	97.4	3	1.3	2	0.9
3. Are the characteristics of the patients included in the study clearly described?	204	88.3	5	2.2	21	9.1
4. Are the interventions of interest clearly described?	128	55.4	39	16.9	63	27.3
5. Are the distributions of principal confounders in each group of subjects to be compared clearly described?	174	75.3	18	7.8	38	16.5
6. Are the main findings of the study clearly described?	221	95.7	3	1.3	6	2.6
7. Does the study provide estimates of the random variability in the data for the main outcomes?	172	74.5	13	5.6	45	19.5
8. Have all the important adverse events that may be a consequence of the intervention been reported?	91	39.4	63	27.3	76	32.9
9. Have the characteristics of patients lost to follow-up been described?	109	47.2	20	8.7	101	43.7
10. Have actual probability values been reported (e.g., .035 rather than < .05) for the main outcomes except where the probability value is less than .001?	167	72.3	14	6.1	49	21.2
Internal validity - confounding						
11. Were the subjects asked to participate in the study representative of the entire population from which they were recruited?	152	65.8	10	4.3	68	29.4
12. Were those subjects who were prepared to participate representative of the entire population from which they were recruited?	143	61.9	15	6.5	72	31.2
13. Were the staff, places, and facilities where the patients were treated representative of the treatment the majority of patients receive?	121	52.4	31	13.4	78	33.8
Internal validity - bias						
14. Was an attempt made to blind study subjects to the intervention they have received?	64	27.7	66	28.6	100	43.3
15. Was an attempt made to blind those measuring the main outcomes of the intervention?	64	27.7	70	30.3	96	41.6
16. If any of the results of the study were based on "data dredging," was this made clear?	90	39.0	85	36.8	55	23.8
17. In trials and cohort studies, do the analyses adjust for different lengths of follow-up of patients, or in case-control studies, is the time period between the intervention and outcome the same for cases and controls?	79	34.2	53	22.9	98	42.4
18. Were the statistical tests used to assess the main outcomes appropriate?	162	70.1	5	2.2	63	27.3
19. Was compliance with the intervention/s reliable?	124	53.7	14	6.1	92	39.8
20. Were the main outcome measures used accurate (valid and reliable)?	174	75.3	7	3.0	49	21.2
Internal validity - confounding (selection bias)						
21. Were the patients in different intervention groups (trials and cohort studies) or were the cases and controls (case-control studies) recruited from the same population?	46	19.9	29	12.6	155	67.1
22. Were study subjects in different intervention groups (trials and cohort studies), or were the cases and controls (case-control studies) recruited over the same period of time?	77	33.3	48	20.8	105	45.5
23. Were study subjects randomized to intervention groups?	88	38.1	47	20.3	95	41.1
24. Was the randomized intervention assignment concealed from both patients and health care staff until recruitment was complete and irrevocable?	77	33.3	57	24.7	96	41.6
25. Was there adequate adjustment for confounding in the analyses from which the main findings were drawn?	137	59.3	19	8.2	74	32.0
26. Were losses of patients to follow-up taken into account?	105	45.5	28	12.1	97	42.0
Power						
27. Did the study have sufficient power to detect a clinically important effect where the probability	125	54.1	25	10.8	80	34.6

### Supplementary document

### Search equations and terms

### Databases

(("Malnutrition/prevention & control*" OR stunting) AND ("infant" OR "child") AND ("prevention" OR "treatment" OR "primary care")) Sort by: Best Match Filters: Infant: birth-23 months ((((Malnutrition/prevention & control* OR stunting OR undernutrition) AND (public-private OR Public Health* OR Public Sector* OR Public-Private Sector Partnerships* OR inter-sectoral OR multi-sectoral)) AND ((partnerships OR collaboration OR consolidation OR cooperation OR planning)) AND (infant OR child)))

## PUBMED

stunting [Title/Abstract] AND (growth disorders [MeSH Terms] AND (treatment[Title/Abstract]

## OVID

(stunting and primary prevention).af. and infant.sh. stunting.af. and primary prevention.sh. and infant.sh. (growth disorders and primary prevention and infant).sh. stunting.at. and primary health care.sh. (growth disorders and primary health care and infant).sh. stunting.af. and growth disorders.sh. and treatment.sh. (stunting and treatment).af. and infant.sh

### Web of Science

TS=(stunting AND "primary prevention")

ALL=growth disorders AND TI=primary prevention

ALL=growth disorders AND ALL=primary prevention AND ALL=stunting

ALL=stunting AND TS= "primary health care" AND TS=infant=

ALL=stunting AND TS=growth disorders AND ALL=treatment AND TS=infant=

### Secondary Sources

### World Health Organization Website

Equation: "desnutrición crónica" OR stunting OR malnutrition) AND (Infant OR child)

Equation: malnutrition stunting intersectoral partnership infant child https://globalnutritionreport.org/reports/global-nutrition-report-2018/three-issues-critical-need-attention/

https://www.who.int/search?page=44&pagesize=10&query=malnutrition%20stunting%20intersectoral%20partnership%20infant%20child&sort=relevance&sortdir=desc&default=AND&f.Countries.size=100&f.Lang.filter=en&f.RegionalSites.size=100&f.Topics.size=100&f.contenttype.size=100&f.doctype.size=101&facet.field=RegionalSites&facet.field=Topics&facet. field=doctype&facet.field=Countries&facet.field=contenttype&facet.field=Lang&tune=true&tune.0=3&tune.1=2&tune.2=2&tune.3=3&tune.4=180&tune.5=75 Accessed: February 27, 2019

### FAO Website

Equation: (("Malnutrition prevention & control*" OR stunting) AND (infant OR child) AND (prevention OR treatment OR "primary care"))

Equation: malnutrition stunting intersectoral partnership infant child http://www.fao.org/search/en/?cx=018170620143701104933%3Aqq82jsfba7w&q=malnutrition+stunting+intersectoral+partnership+infant+child&cof=FORID%3A9&siteurl=www.fao.org%2Fhome%2Fen%2F&ref=&ss=0j0j1 Accessed: February 27, 2019

### World Bank Website

Equation: (("Malnutrition prevention & control*" OR stunting) AND (infant OR child) AND (prevention OR treatment OR "primary care")) Equation: ((((stunting) AND (inter-sectoral OR multi-sectoral) AND (infant OR child))) http://www.worldbank.org/en/search?q=%28%28%E2%80%9CMalnutrition+prevention+%26+control*%E2%80%9D+OR+stunting%29+AND+%28infant+O R+child%29+AND+%28prevention+OR+treatment+OR+%E2%80%9Cprimary+care%E2%80%9D%29%29

http://www.worldbank.org/en/search?q=%28%28%28%28stunting%29+AND+%28inter-sectoral+OR+multi-sectoral%29+AND+%28infant+OR+child%29%29%29&currentTab=1 Accessed: February 27, 2019

### UNICEF Website

Equation: (("Malnutrition prevention & control*" OR stunting) AND (infant OR child) AND (prevention OR treatment OR "primary care")) Equation: malnutrition stunting intersectoral partnership infant child https://www.unicef.org/search?force=0&query=malnutrition+stunting+intersectoral+partnership+infant+child&search_date_range_picker=&changed%5Bmin%5D=&changed%5Bmax%5D= Accessed: February 27, 2019

### GOOGLE and Colombian National Websites

### Ministerio de Salud y Secretaría de Salud

https://www.minsalud.gov.co/Paginas/default.aspx

Keywords: Stunting; Retraso en talla; Desnutrición crónica; Malnutrición Accessed: February 27, 2019

### Instituto Colombiano de Bienestar Familiar - ICBF

https://www.icbf.gov.co/

Key words: Stunting; Retraso en talla; Desnutrición crónica; Malnutrición Accessed: February 27, 2019

### Secretaría Distrital de Integración Social

http://www.integracionsocial.gov.co/ Keywords: Desnutrición crónica. Accessed: February 27, 2019
